# Supplementary material for: Chitosan Films Loaded with Alginate Nanoparticles for Gentamicin Release on Demand
Source: Polymers (Basel). 2025 Aug 21;17(16):2261. doi: 10.3390/polym17162261 (PMC12390268; doi:10.3390/polym17162261)
Supplement: Supplementary file 1 [file polymers-17-02261-s001.zip › polymers-3757772-supplementary.pdf]

Article

Chitosan films loaded with alginate nanoparticles for gentamicin release on-demand

Cecilia Zorzi Bueno [1]*, Helton José Wiggers [1], Pascale Chevallier [2], Francesco Copes [2] and Diego Mantovani [1,2]*

1 Laboratory for Biomaterials and Bioengineering (LBB-BPK), Associação de Ensino, Pesquisa e Extensão BIOPARK, Max Planck Avenue, 3797, Building Charles Darwin, Toledo 85919-899, PR, Brazil; cecilia.bueno@bpkedu.com.br, helton.wiggers@bpkedu.com.br
2 Laboratory for Biomaterials and Bioengineering (LBB-UL), Canada Research Chair Tier I, Department of Min-Met-Materials Engineering & CHU de Quebec Research Center, Division Regenerative Medicine, Laval University, Quebec City, QC G1V0A6, Canada; pascale.chevallier@crchudequebec.ulaval.ca, francesco.copes.1@ulaval.ca, diego.mantovani@gmn.ulaval.ca
* Correspondence: cecilia.bueno@bpkedu.com.br & diego.mantovani@gmn.ulaval.ca

Supplementary Information

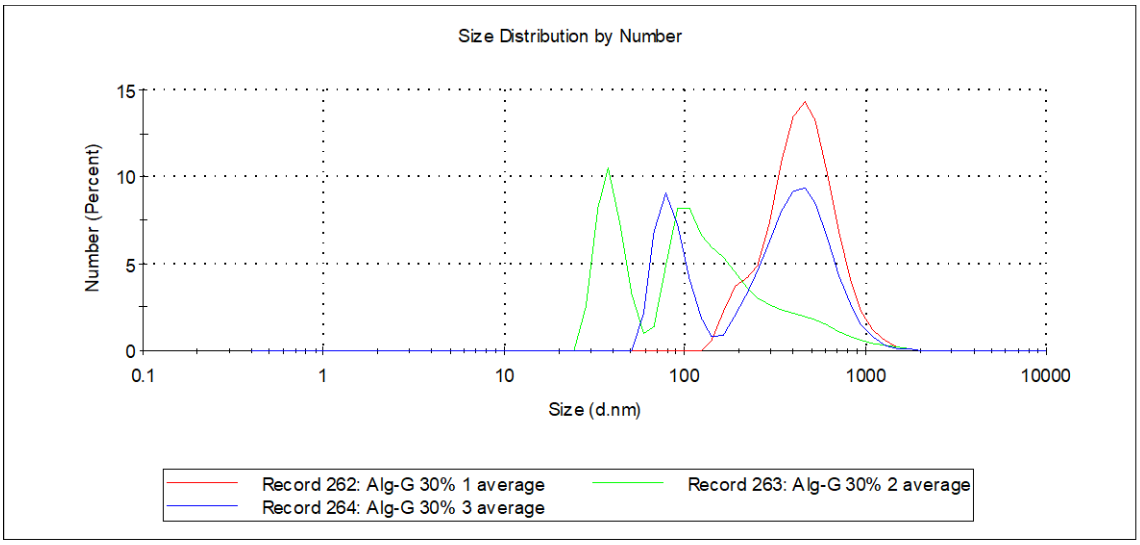

Figure S1. Dynamic light scattering analysis of alginate-gentamicin nanoparticles prepared with gentamicin at 30% w/w.

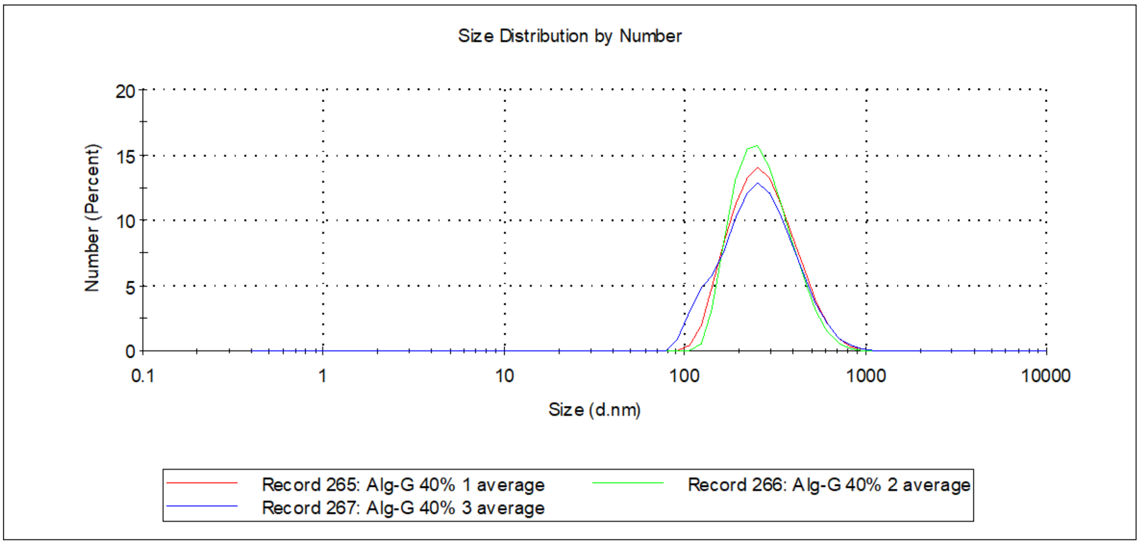

Figure S2. Dynamic light scattering analysis of alginate-gentamicin nanoparticles prepared with gentamicin at 40% w/w.

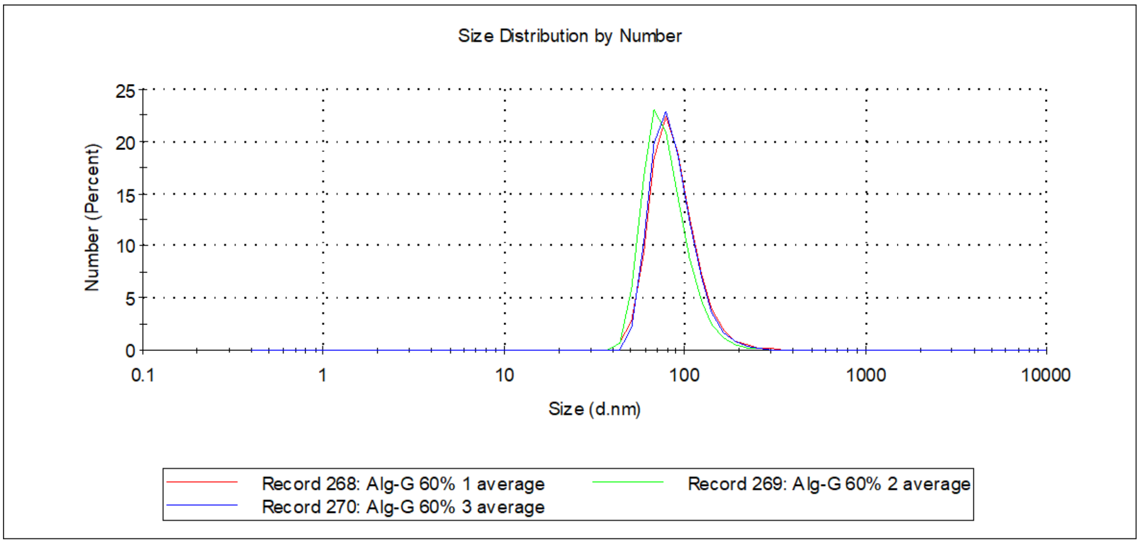

Figure S3. Dynamic light scattering analysis of alginate-gentamicin nanoparticles prepared with gentamicin at 60% w/w.

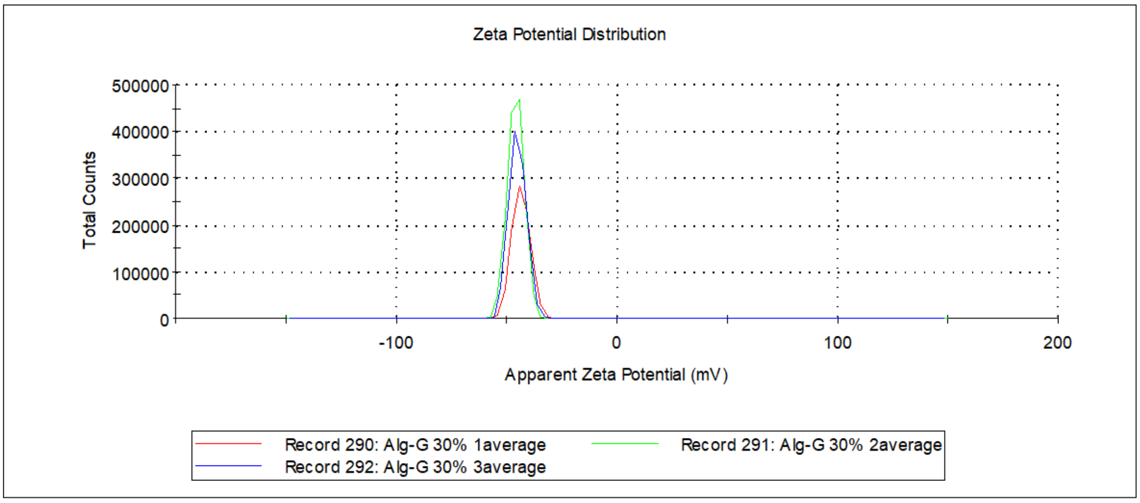

Figure S4. Zeta potential analysis of alginate-gentamicin nanoparticles prepared with gentamicin at 30% w/w.

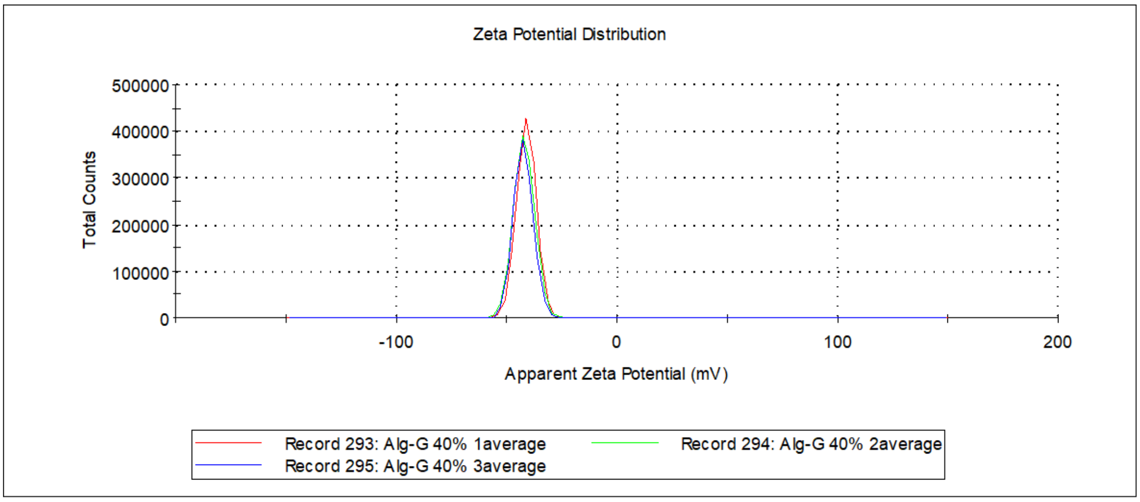

Figure S5. Zeta potential analysis of alginate-gentamicin nanoparticles prepared with gentamicin at 40% w/w.

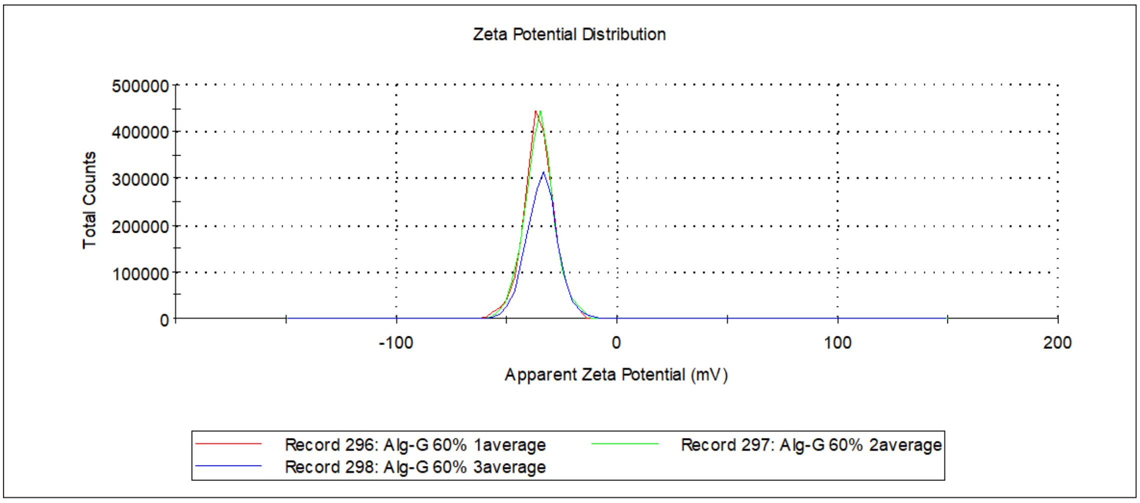

Figure S6. Zeta potential analysis of alginate-gentamicin nanoparticles prepared with gentamicin at 60% w/w.
